# Efficacy of beta-blocker therapy in symptomatic athletes with exercise-induced intra-ventricular gradients

**DOI:** 10.1186/1476-7120-8-38

**Published:** 2010-09-02

**Authors:** Carlos Cotrim, Luís R Lopes, Ana R Almeida, Rita Miranda, Almeida G Ana, Hortense Cotrim, José P Andrade, Eugenio Picano, Manuel Carrageta

**Affiliations:** 1 Cardiology Department, Garcia de Orta Hospital, Almada, Portugal; 2 Cardiology Department, Santa Maria Hospital, Lisboa, Portugal; 3 Health School Egas Moniz, Instituto Superior de Saúde Egas Moniz, Caparica, Portugal; 4 Institute of Clinical Physiology of CNR, Pisa, Italy; 5 Lisboa University Medical School, Lisboa, Portugal

## Abstract

**Background:**

Upright exercise stress echocardiography (SE) induces significant intraventricular gradient (IVG) and systolic anterior motion (SAM) in a large proportion of symptomatic athletes, who may therefore benefit from a negative inotropic therapy.

The purpose of the present study was to assess the effect of chronic oral β blocker therapy on the occurrence of exercise-induced IVG and mitral valve SAM, in symptomatic athletes.

**Methods:**

We enrolled 35 symptomatic athletes (age = 23 ± 11 years) with IVG (>30 mmHg) during SE off therapy. All repeated SE on chronic oral beta-blocker therapy (atenolol up to 50 mg, bisoprolol up to 10 mg, or metoprolol up to 100 mg daily according to physician-driven choice).

**Results:**

On therapy, there was during SE a reduction in IVG (35 off vs 17 on beta blocker, p < 0.01), decrease of IVG (102 ± 34 mmHg off vs 69 ± 24 mmHg on beta blocker, p < 0.01), peak heart rate (178 ± 15 bpm off vs 157 ± 9 bpm on beta blocker), SAM (24 off vs 9 on beta blocker, p < 0.001), symptoms during SE (17 off vs 2 on beta blocker p < 0.001), ST segment depression (13 off vs 2 on beta blocker, p < 0.001).

**Conclusions:**

In athletes with positive screening on medical evaluation for sports practice and IVG on exertion, treatment with oral beta blockers improved symptoms in the large majority of patients. Symptomatic benefit was mirrored by objective evidence of improvement of echocardiographic signs of obstruction (IVG and SAM) and reduction of ischemia-like electrocardiographic changes.

## Introduction

Upright exercise stress echocardiography (SE) induces a significant intraventricular gradient (IVG) in a large population of symptomatic athletes [1], but the clinical implications of this finding remain unclear. Since the underlying pathophysiological condition is an hyperkinetic response of the left ventricle, there is rationale for a targeted negative inotropic therapy with beta-blockers in these subjects, as it has been previously described in Syndrome X patients [2]. However, the evidence supporting the initiation, driven by SE results, of a beta-blocker therapy in athletes is anecdotal to date. The aim of the present open-label, prospective, non-randomized study was to provide proof of concept that SE can be a guide to tailored treatment in athletes with positive screening [3] on medical evaluation for sports practice, that develop IVG and mitral valve SAM on exertion.

## Methods

### Population

We performed SE in 139 A (135 amatheur), 30 (22%) of whom were female, mean aged 22 ± 9.9 years old (9 to 56), with a positive screening for sports practice but with normal rest echocardiogram. These athletes were referred to our center between 2002 and 2010, after the initial publication [4] of a case report of a runner, who had a normal rest echocardiogram and normal coronary arteries and was submitted to an exercise stress test, during which had developed ischemia and IVG. In 52 (37,4%) of these athletes, SE disclosed IVG with a mean end-systolic peak of 95 ± 35 mmHg (50 to 193), combined with mitral valve SAM in 33 of them. The 35 pts who repeated the SE under treatment with β blockers are the study group. Two (6%) of them were women. Mean age was 23 ± 11 years old (11 to 56). Thirty three had exercise related symptoms or positive exercise electrocardiography.

Exclusion criteria were the presence of left ventricular (LV) hypertrophy, mitral valve prolapse or other valvular disease. At the time of the second SE assessment all athletes were medicated with beta-blockers. In all cases, beta-blockers were taken at breakfast on the morning of the exam, and had been prescribed by the patients' physicians: metoprolol 100 mg (in two athletes), atenolol 50 mg in one athlete and bisoprolol in 32 athletes (2.5 mg in two, 10 mg in one and 5 mg in 29 athletes). Bisoprolol was started with 1.25 mg once daily in pediatric age and with 2.5 mg in the adults and was titrated to the maximum tolerated dose or until clinical benefit was seen.

The study protocol was approved by the Ethics Committee at Garcia de Orta Hospital and conforms to the ethical guidelines of the 1975 Declaration of Helsinki. All athletes or parents gave informed consent for the study.

### Exercise stress echocardiography

A complete echocardiographic assessment was performed, including measurement of the LV outflow tract and calculation of the index, LV mass index, relative wall thickness, and LV end-diastolic volume.

Maximum flow velocity was also assessed by continuous wave Doppler in the LV outflow tract in five-chamber apical view to calculate the intra-ventricular gradient.

The athletes had previously undergone exercise stress echocardiography without beta-blockers, and subsequently repeated the exam under beta-blocker therapy during the following year. The methodology used [5] included assessment of wall motion during treadmill exercise, as well as of flows by pulsed and continuous wave Doppler for detection of IVG and color Doppler. Mitral valve motion is also assessed, particularly for SAM. The exams were recorded on video in their entirety, with parts recorded on optical disk.

A significant intra-ventricular gradient was considered if greater then 30 mmHg during/immediately after stress, at the end of systole.

The Doppler echocardiographic parameters presented are the mean of three measurements obtained on consecutive, good-quality recordings.

### Ergometric parameters

The following parameters were assessed during SE: test duration (in seconds), systolic blood pressure at rest and at peak exercise, heart rate at rest and at peak exercise, peak double product, and occurrence of ST segment alterations - ST depression of 1 mm 80 ms after the J point. Occurrence of symptoms during the test similar to those that prompted the patients' original assessment was also recorded.

### Statistical analysis

The results are presented as means ± standard deviation for continuous variables, and frequencies and percentages for categorical variables. The comparison between the two assessments was performed by using the Student's t test for continuous variables and the McNemar test for paired samples for nominal qualitative data.

## Results

Of the 35 athletes enrolled in the study, two (6%) were women. Mean aged was 23 ± 11 years old (11 to 56). Fifteen athletes practice athletics, eleven practice soccer, and the other nine, other sports - diving (1), tennis (1), basketball (1), swimming (2), rowing (1), handball (2), karate (1). The initial evaluation was done due to dizziness in fourteen athletes, chest pain in eight athletes, palpitations in four athletes, exercise stress electrocardiography with ST alterations in seven athletes, ECG repolarization alterations in one athlete and documented ventricular premature complexes in one athlete. On the resting echocardiogram, at the first assessment, LV outflow tract index was 10.6 ± 0.9 mm/m2, IVS index was 5.2 ± 0.67 mm/m^2^, PW index was 4.73 ± 0.47 mm/m^2^, LV end-diastolic diameter index was 26.21 ± 1.96 mm/m^2^, LV mass index was 76.6 ± 10.6 g/m2, relative wall thickness was 0.36 ± 0.03, and LV end-diastolic volume was 46.3 ± 7.3 ml/m^2^. No wall motion abnormalities were detected in any of the exams, with or without beta blockers. IVG at peak exercise on the first assessment was 102 ± 34 mmHg. Thirty athletes (85%) showed improvement and are doing well at the moment of the second SE. The main results of the variables studied are shown in Table 1. The inclusion criterion was the presence of IVG in the first SE. The evaluation by Doppler echocardiography was feasible in the whole study population. We should note that the Doppler evaluation was done during/immediately after stress, in orthostatic position and then in left lateral decubitus. The Doppler evaluation was possible during exercise (at peak) in 33 (94%) and in all the athletes immediately after stress.

**Table 1:** Variables assessed in the two stress echocardiograms

	SE1	SE2	p
Test duration (sec)	762 ± 107	750 ± 86	0,236

Peak exercise HR (bpm)	179 ± 15	157 ± 9	<0,001

Peak exercise SBP (mmHg)	164 ± 20	155 ± 19	<0,001

Peak exercise DP (HRxSBPPeak)	29424 ± 3746	22798 ± 16170	<0,001

Symptoms during SE (n)	17/35 (49%)	2/35(5.5%)	<0.001

Peak IVG (n)	35/35(100%)	17/35(49%)	<0.001

SAM (n)	24/35 (69%)	9/35 (26%)	<0.001

ST - segment alterations (n)	13/35 (37%)	2/35(5.7%)	<0.001

DP: double product; HR: heart rate; IVG: intra-ventricular gradient; SAM: mitral valve systolic anterior motion; SBP: systolic blood pressure; SE: stress echocardiography

## Discussion

Intra-ventricular gradients in a symptomatic patient were first described by Lau et al [6], who detected the phenomenon in a patient with effort angina and treated it with bisoprolol, resulting in clinical improvement and a significant reduction in the IVG.

Subsequently, we also demonstrated the presence of IVG in patients with angina and angiographically normal coronary arteries [4] which led to a study of 91 syndrome X patients [7], among whom we selected 20 patients, with IVG, that were treated with beta blocker, with symptomatic benefit [2].

Particular problems arise when treating athletes in pediatric age - as were 15 (43%) athletes of this study - because beta blockers are used following extrapolation from data obtained with studies in adults. The most obvious problem is that the dose range will not have been established during drug development. The laws and the regulatory processes that govern the pharmaceutical industry have historically led to exclusion of children from the drug development process. However, clinical benefit of beta blockers use, in children with left ventricular outflow obstructive pathology, has been shown [8,9]. The benefit of beta blockers in the treatment of symptoms possibly related to IVG elicited by exercise, in patients without hypertrophic cardiomyopathy [2,10,11], has been showed before and so we decided to use them also in children.

**Figure 1:**
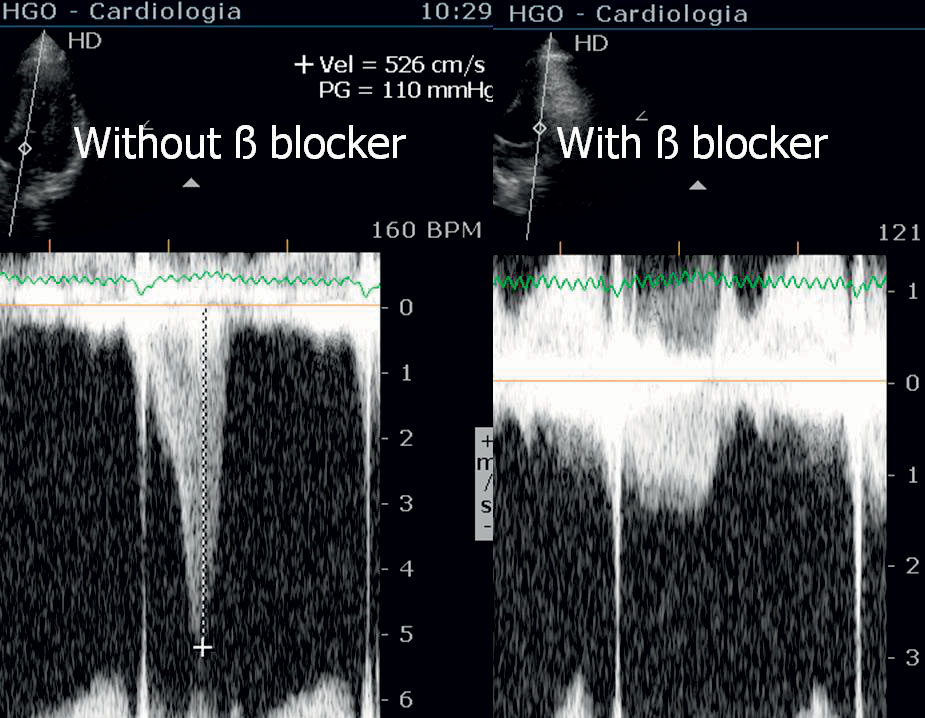
IVG in one athlete assessed before (left) and after (right) - beta blocker therapy

In our study we found that beta blockers reduced heart rate, ST-segment alterations during exercise, systolic blood pressure and hence peak HRxSBP. The occurrence of IVG (Figure 1) and SAM (Additional file 1) on exertion also decreased significantly ((Additional file 2). These changes were accompanied by improvement in symptoms during exercise testing and follow-up.

In the present study there was improvement in symptoms, however the functional capacity continued the same. This fact favors the present legislation that doesn't consider illegal the use of beta blockers in the majority of sports practiced by the athletes that were included [12].

The relation between IVG and symptoms or ST alterations during exercise stress electrocardiography has been demonstrated previously [1,4,6,7]. Is also of importance the fact that the two studies [13,14] that searched IVG in healthy people have failed to found them. This fact reinforces the probable pathologic significance of exercise induced IVG [15].

The results of this study suggest that exercise stress echocardiography in orthostatic position in treadmill would help in selecting symptomatic athletes with positive screening on medical examination for sports practice, who develop IVG on exertion and who would respond particularly well to these drugs. A randomized study is needed to demonstrate that our practice of searching for IVG, and treatment with beta blockers, in athletes with positive screening should be generalized in clinical practice.

It is established [16] that semi-supine exercise echocardiography is more feasible than treadmill however to the best of our knowledge there is no study directly comparing the two methods for the evaluation of the same patients and the same Doppler parameters.

## Study Limitations

One limitation of the study is the relatively small sample size. However, the 35 patients met stringent inclusion criteria, since all of them were symptomatic athletes with objective evidence of IVG during exercise, and therefore represent a selected cohort which is the most likely candidate to benefit from a targeted therapy with beta-blockers. As a further limitation, we did not perform a randomization with double-blinding of subjects and investigators for obvious ethical, feasibility and cost reasons. In our open-label design, both athletes and investigators were aware of the therapy in progress, and beta-blocker type and dosage varied widely reflecting the garden variety of treatment in the real world. In addition, all tests under therapy were performed at the second assessment, which may have per se influenced the results, particularly treadmill test duration and how the athletes rated their symptoms. However, this would not influence objective parameters such as the presence and severity of IVG, which was the study primary endpoint. Further studies, preferably placebo-controlled randomized trials, are needed at this point in order to assess conclusively the potential benefits for beta blockers use in these athletes. The present lack of recommendations for the searching of IVG during exercise in athletes with positive screening and about the treatment of these patients with beta blockers further underscore the importance of additional studies.

## Conclusions

In athletes with positive screening - mostly by symptoms - on medical evaluation for sports practice and IVG on exertion, treatment with oral β blockers prevented the occurrence of IVG and SAM or significantly reduced its magnitude. These changes were associated to significant reduction in heart rate at peak exercise and mirrored clinical improvement occurring in 85% of the study population.

Exercise echocardiography represents a useful tool to evaluate athletes with positive screening and normal rest echocardiogram and may permit to select them for beta blocker therapy. The finding of this study represents a proof-of-concept of possibility to tailor effective beta-blocker therapy, driven by exercise-Doppler echocardiography results, in symptomatic athletes.

## Competing interests

The authors declare that they have no competing interests.

## Authors' contributions

CC planned the study, reviewed literature and wrote the manuscript, performed exercise echocardiography, and performed clinical assessment of the athletes. LRL, ARA and RM performed clinical assessment of the athletes, participated in drafting the article, and revised the manuscript for important intellectual content. AGA participated in planning the study and revised the manuscript for important intellectual content. HC participated in planning and monitoring the study according to the best ethical practices of investigation in children. JPA performed clinical assessment of the athletes and revised the manuscript for important intellectual content. EP participated in planning the study, data analysis, drafting the manuscript, and revising the manuscript for important intellectual content. MC participated in planning the study and revised the manuscript for important intellectual content. All authors read and approve the final manuscript.

## Supplementary Material

Additional file 1 **Echocardiographic images obtained at peak exercise in first SE in one athlete of the study, without treatment**. Apical four and five chamber view obtained in apical window during exercise containing two dimensional data with SAM of mitral valve Click here for file

Additional file 2 **Echocardiographic images obtained at peak exercise in the second SE in one athlete of the study, under treatment with beta blocker**. Apical four and five chamber view obtained in apical window during exercise containing two dimensional data without SAM of mitral valve Click here for file
